# Uncovering microstructural architecture from histology

**DOI:** 10.1101/2024.03.26.586745

**Published:** 2024-03-29

**Authors:** Marios Georgiadis, Franca auf der Heiden, Hamed Abbasi, Loes Ettema, Jeffrey Nirschl, Hossein Moein Taghavi, Moe Wakatsuki, Andy Liu, William Hai Dang Ho, Mackenzie Carlson, Michail Doukas, Sjors A. Koppes, Stijn Keereweer, Raymond A. Sobel, Kawin Setsompop, Congyu Liao, Katrin Amunts, Markus Axer, Michael Zeineh, Miriam Menzel

**Affiliations:** 1 Department of Radiology, Stanford University; Stanford, 94305, USA.; 2 Institute of Neuroscience and Medicine (INM-1), Forschungszentrum Jülich GmbH; 52425 Jülich, Germany.; 3 Department of Imaging Physics, Faculty of Applied Sciences, Delft University of Technology; 2628 CJ Delft, the Netherlands.; 4 Department of Otorhinolaryngology and Head and Neck Surgery, Erasmus MC, University Medical Center Rotterdam; 3015 CN Rotterdam, the Netherlands.; 5 Department of Pathology, Stanford University; Stanford, 94305, USA.; 6 Department of Neurology and Neurological Sciences, Stanford University; Stanford, 94305, USA.; 7 Department of Pathology, Erasmus MC, University Medical Center Rotterdam; 3015 CN Rotterdam, the Netherlands.; 8 C. and O. Vogt Institute for Brain Research, University Hospital Düsseldorf, Medical Faculty, University Düsseldorf, Germany.; 9 Department of Physics, School of Mathematics and Natural Sciences, University of Wuppertal; 52119 Wuppertal, Germany.

## Abstract

Microstructural tissue organization underlies the complex connectivity of the brain and controls properties of connective, muscle, and epithelial tissue. However, discerning microstructural architecture with high resolution for large fields of view remains prohibitive. We address this challenge with computational scattered light imaging (ComSLI), which exploits the anisotropic light scattering of aligned structures. Using a rotating lightsource and a high-resolution camera, ComSLI determines fiber architecture with micrometer resolution from histological sections across preparation and staining protocols. We show complex fiber architecture in brain and non-brain sections, including histological paraffin-embedded sections with various stains, and demonstrate its applicability on animal and human tissue, including disease cases with altered microstructure. ComSLI opens new avenues for investigating fiber architecture in new and archived sections across organisms, tissues, and diseases.

## One Sentence Summary

We uncover microstructural architecture of new or archived human and animal histological sections in health and disease.

The microstructural organization of tissues serves their function and imparts their properties. In the brain, complex neuronal networks enable brain connectivity, and mapping all neuronal trajectories is a major goal of neuroscience (1, 2). Beyond the brain, fiber orientation controls the mechanical properties and is disturbed in disease in connective (3, 4), epithelial (5, 6), and muscle (7) tissues. Nanometer-resolution methods like electron microscopy achieve exquisite results in small volumes of various tissues (8–10). 3D-polarized light imaging (3D-PLI) and scattered light imaging (SLI) have delivered micron-resolution maps in unstained brain sections (11–16). While 3D-PLI shows specific sensitivity to myelination and allows tracing fiber tracts from deep white matter into the depth of the cerebral cortex, SLI provides superior insights into complex fiber tract crossings. Tomographically, synchrotron X-ray scattering and diffusion magnetic resonance imaging (dMRI) can retrieve fiber orientations in the musculoskeletal (17, 18) or nervous (19, 20) system, but with resolutions down to tens of micrometers they cannot resolve single fibers, e.g., individual nerve axons, and require microscopic validation (21). Thus, there is a need for an easily accessible, widely applicable micron-resolution method to elucidate tissue microstructural organization.

At the same time, the most common method for pathological evaluation of animal and human tissue is paraffin embedding and microtome sectioning followed by histological staining (22). As a result, thousands of formalin-fixed paraffin-embedded (FFPE) tissue sections are produced daily, adding to the millions already archived.

We here present the use of computational scattered light imaging (ComSLI), a fast and cost-effective optical microscopy method, to reveal the microscopic fiber orientations of virtually any new or archived FFPE section from animal or human, healthy or diseased tissue, also in regions with highly interwoven fibers. Using ComSLI, we recover orientation maps from nervous, muscle, and bone tissue sections, prepared using different protocols, stains, thicknesses, and storage periods. This approach enables retrospective investigations of histology slides, combining information from any stain with fiber orientations, and can potentially help reveal new micro-organization patterns as well as relationships between the microstructure and the underlying biology in healthy and diseased, animal and human tissues.

## Retrieving microscopic fiber orientations from whole human brain histology sections

**Fig. 1.**
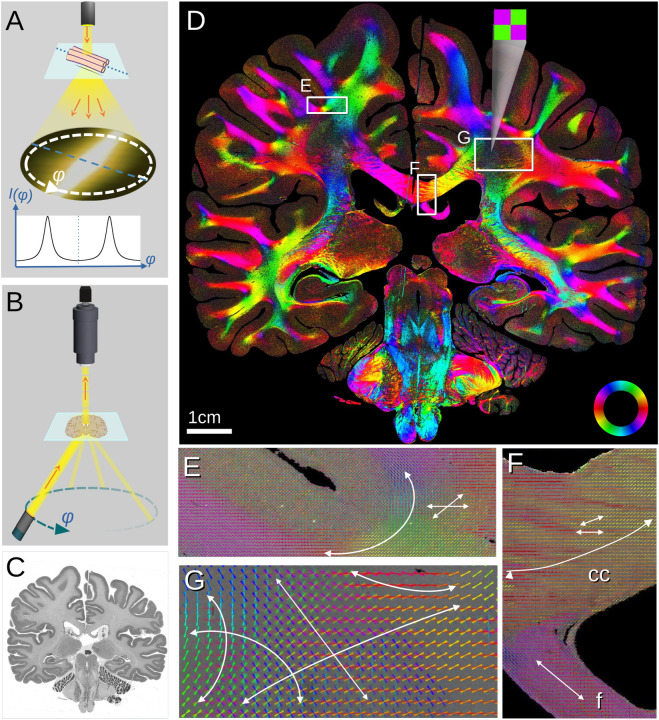
Retrieving fiber orientations from a whole human brain section. (**A**) Light beam scattered by in-plane fiber bundle, predominantly perpendicular to the fibers, resulting in paired peaks in the azimuthal intensity profile, I(*φ*), whose midline indicates the fiber orientation. (**B**) Schematic of ComSLI measurement setup, allowing to measure the azimuthal intensity profile by reversing the light path. The tissue section is illuminated at ~45° elevation angle by a rotating lightsource while a camera captures a high-resolution image of the section at each (azimuthal) rotation angle *φ*. (**C**) Brightfield microscopy image of a coronal human brain section (second *BigBrain* dataset, section no. 3452, FFPE, silver-stained), 1μm/pixel. (**D**) Fiber orientation map of the brain section, 7μm/pixel. The fiber orientations are shown as multi-colored pixels according to the colorwheel in the bottom right; multiple colors per pixel indicate multiple fiber orientations, such as the zoomed-in green-purple pixel of the right corona radiata. (**E-G**) Fiber orientations for boxes marked in (D). Orientations are encoded both by the line direction and its color. (**E**) U-fiber. (**F**) Corpus callosum (cc) and fornix (f). (**G**) Right corona radiata. For better visualization, the orientation-depicting lines of sets of 15×15 pixels (E-F) and 75×75 pixels (G) were overlaid.

Oriented structures such as nerve fiber bundles scatter light predominantly perpendicular to their main axis (23), yielding characteristic light intensity profiles, I(*φ*), where the mid-position of a peak pair indicates the fiber orientation (Fig. 1A). ComSLI reverses the light path, which is physically equivalent, to determine the orientations of fibers contained in each micrometer-sized image pixel. The setup (Fig. 1B) consists of a high-resolution camera with small-acceptance-angle lens, and a rotating LED-lightsource illuminating the tissue section from below at ~45° elevation. The camera captures images of the sample at different lightsource rotation angles *φ*, yielding an image series in which each pixel contains a light intensity profile across different illumination angles, I(*φ*), according to the orientation of its contained fibers (Movie S1). Analyzing the peaks in each intensity profile yields multiple fiber orientations for each image pixel.

We first hypothesized that using ComSLI we can retrieve fiber orientations with micrometer resolution from extended field-of-view sections, such as entire FFPE human brain sections (*Jülich BigBrain* (24)), Fig. 1C. Orientation information was recovered from single and crossing fiber bundles in each pixel, in both white and gray matter (Fig. 1D). Microscopic details become apparent: U-fibers connecting the precentral gyrus to neighboring gyri, Fig. 1E, with subjacent deeper fibers crossing the main tracts (small arrows). Fibers in the corpus callosum (cc) are relatively homogeneously decussating between the two hemispheres with small angular variations (Fig. 1F, small arrows), while fibers of the fornix (f) course superomedially, and complex fiber crossings in dense corona radiata white matter are discerned (Fig. 1G).

Such investigations at whole-brain level unveil microscopic fiber orientations, enabling detailed investigation of anatomy, correlations with cellular information, or pathology.

## Fiber architecture can be retrieved for various stains, preparation protocols, and species

Next, we tested whether ComSLI can retrieve information from histological sections independent of stain, sample preparation, and species, since the orientation of scattered light is affected solely by the orientation of scattering fibers.

**Fig. 2.**
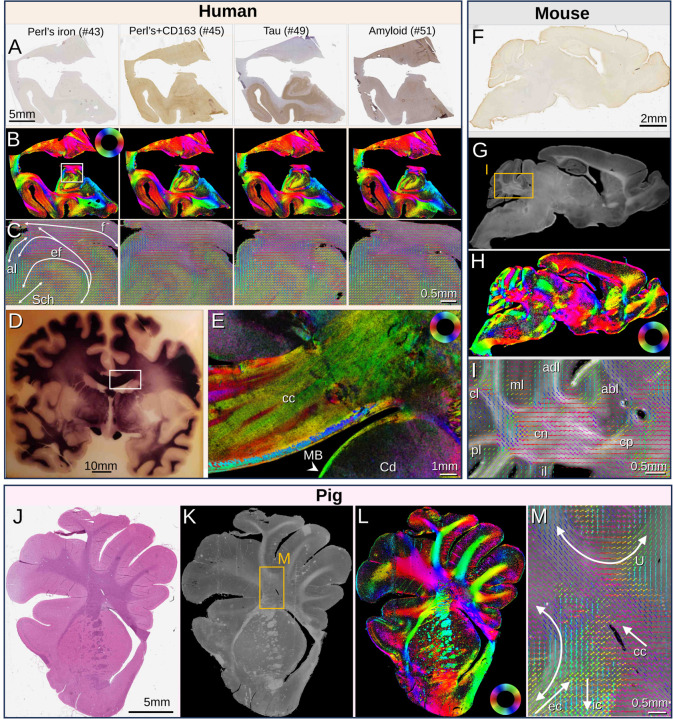
ComSLI detects fiber orientations independent of tissue preparation and species. (**A-C**) **Human Hippocampi**, FFPE sections with different stains. (**A**) Brightfield microscopy images, 0.5μm/pixel. (**B**) ComSLI fiber orientation maps, 7μm/pixel, orientations encoded by colorwheel. (**C**) Zoomed-in fiber orientation vector maps (white box in (B)), including parts of cornu ammonis and multiple tracts: fornix (f), endfolial pathway (ef), alveus (al), Schaffer collaterals (Sch). Vectors of 15×15 pixels are overlaid. (**D-E**) **Myelin-stained human brain section (~**120-year-old). (**D**) Section photograph. (**E**) ComSLI color-coded fiber orientation map (3μm/pixel) of marked region in (D), cc: corpus callosum, Cd: Caudate nucleus, MB: Muratoff bundle (arrowhead). (**F-I**) **Sagittal mouse brain section**, FFPE, microglia-stained. (**F**) Brightfield image, 0.5μm/pixel. (**G**) ComSLI average scattering signal, 4.25μm/pixel. (**H**) Fiber orientation map. (**I**) Zoom-in of box in (G) (cerebellar area), including cerebellar peduncle (cp), cerebellar nuclei (cn), lobes (inferior-il, posterior-pl, central-cl, anterodorsal-adl, anterobasal-abl), and gray matter including oriented molecular layer (ml) fibers. Vectors overlaid for 15×15 pixels. (**J-M**) **Pig hemisphere-brain section**, FFPE, H&E-stained. (**J**) Brightfield image, 0.5μm/pixel. (**K**) ComSLI average scattering signal, 9μm/pixel. (**L**) Fiber orientation map. (**M**) Fiber orientations from box in (K), overlaid for 15×15 pixels, including U-fiber tracts (curved arrows) and multiple crossing fibers (straight arrows); external capsule (ec), internal capsule (ic), corpus callosum (cc).

We scanned consecutive FFPE sections of a human hippocampus with various stains, colors, intensities, antibody concentrations, and counter-stains (see Methods). Figures 2 and S1 show the brightfield images (A), fiber orientation maps (B), and zoomed-in views of the cornu ammonis and multiple fiber tracts (C). Despite the different staining protocols, targets, intensities, and counter-stains, the retrieved fiber orientations are almost identical across hippocampal sections, with small anatomical differences between sections.

We next investigated a section embedded in celloidin, myelin-stained approximately 120 years ago, from the brain collection of the Cécile and Oskar Vogt Institute for Brain Research (Fig. 2D–E): Despite the section age and the low light transmission in parts of the section (Fig. 2D), the individual nerve fiber orientations were retrieved (Fig. 2E), e.g., in the corpus callosum and the Muratoff bundle (subcallosal fasciculus) running subependymal to the caudate nucleus surface (25) (arrowhead).

This led us to ask (i) if we can also retrieve orientations from sections at different steps of sample preparation, and (ii) if the sample processing affects the method’s sensitivity. We scanned two sections in paraffin just after sectioning, deparaffinization, and staining (fig. S2). Excitingly, fiber orientations could be visualized from sections still in paraffin (fig. S2A–B), as well as from completely unstained sections (fig. S2C–D), showing similar results as the stained sections (fig. S2E–F). The derivation and concordance of white and gray matter orientations across sample preparation stages highlight the wide range of histology sections that can be analyzed with ComSLI.

To confirm the technique works across species, we measured multiple animal brain sections with different stains, including from mouse (Fig. 2F–I) and pig (Fig. 2J–M). Tracts were retrieved with micrometer resolution, such as the intricate mouse cerebellar connectivity (Fig. 2I) and multiple crossing and U-fibers in the pig corona radiata (Fig. 2M).

## Microscopic fiber orientations provide unique information in pathology

To see if ComSLI can unveil disturbed microscopic fiber orientations and provide unique information in pathologic human tissue, we studied human brain samples with neuropathology.

**Fig. 3.**
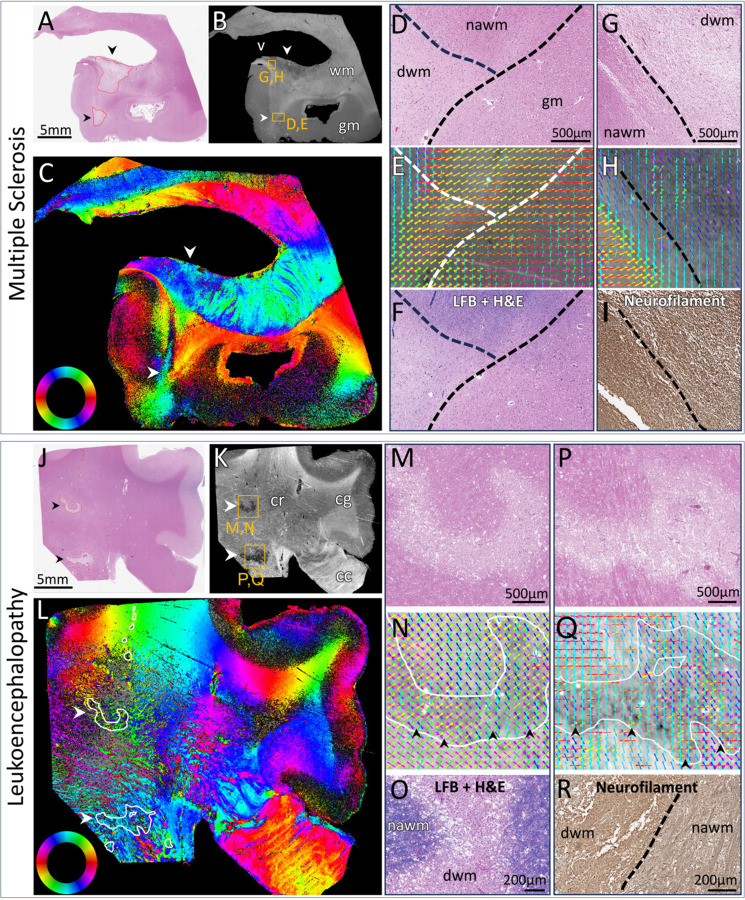
Human brain pathology measured with ComSLI (FFPE sections, H&E-, LFB-, and neurofilament-stained). Brightfield microscopy: 0.5μm/pixel size, ComSLI: 9μm/pixel. (**A-I**) **Multiple sclerosis (MS) brain**. (**A**) Brightfield image; white matter lesions (red contours) indicated by arrowheads. (**B**) Average scattering signal from ComSLI (v: ventricle, gm: gray matter, wm: white matter). (**C**) Fiber orientation map. (**D**) Zoom-in of brightfield H&E image corresponding to orange box in (B), at the interface of normal appearing white matter (nawm), demyelinated white matter (dwm), and gray matter (gm). (**E**) Fiber orientations in the same area. 15×15 pixel orientations are overlaid for visual clarity. (**F**) The same region in a consecutive LFB+H&E-stained section. (**G**) Zoom-in of brightfield H&E image corresponding to orange box in (B). (**H**) Fiber orientations in the same area. (**I**) The same region in a consecutive neurofilament-stained section. (**J-R**) **Leukoencephalopathy brain**. (**J**) Brightfield image; lesions are annotated with red contour, larger lesions indicated by arrowheads. (**K**) Average scattering signal. cc: corpus callosum, cg: cingulum, cr: corona radiata. (**L**) Fiber orientation map, lesions annotated with white contour. (**M**) Zoom-in of a large lesion in the H&E image corresponding to upper orange box in (K). (**N**) ComSLI fiber orientations in the same area, showing some less aligned fibers (arrowheads). (**O**) Zoom-in of a lesion in a myelin-stained LFB+H&E consecutive section, showing the dramatic loss of myelin in the lesion as well as axonal loss and spongiosis, causing the reduced ComSLI signal. (**P**) Zoom-in of the H&E image corresponding to bottom orange box in (K). (**Q**) ComSLI fiber orientations in the same area, showing some areas of less aligned fibers (arrows). (**R**) Zoom-in of a lesion in a neurofilament-stained consecutive section.

First, we investigated a brain of a subject with multiple sclerosis (MS) – a neurological autoimmune disease damaging the insulating myelin sheaths of nerve fibers and impairing signal transmission. We imaged a section with focal demyelination (Fig. 3A, red-annotated lesions). To fully characterize the lesion pathologically, we performed additional myelin (Luxol fast blue - LFB) and neurofilament stains in consecutive sections (fig. S3). The light scattering signal (Fig. 3B) was not affected by myelin loss within the red-outlined lesion. The axonal orientation could be retrieved even in regions of extensive demyelination (Fig. 3C, arrowheads). In regions with a transition between normal-appearing white matter (nawm) and demyelinated white matter (dwm) axons (Fig. 3D–F), the ComSLI signal intensity is preserved and the fiber orientations are continuing their course despite the demyelination. This highlights the robustness of the method, which is not dependent on the presence of myelin, but rather of axonal orientations. Although the ComSLI signal is not significantly affected by myelin, it detects changes in tissue density; the lower ComSLI signal periventricularly (Fig. 3B, upper arrowhead) is due to the lower neuropil density in the region (Fig. 3G,I), with less corresponding fiber organization present (Fig. 3H). Even then, ComSLI retrieves fiber orientations from the remaining axons (Fig. 3H).

Next, we studied a brain sample with necrotizing leukoencephalopathy (LE) which is characterized by white matter necrosis with loss of neurons and myelin. The pathology-confirmed lesions (red-contoured areas in Fig. 3J) show a lower scattering signal (Fig. 3K), and are clearly delineated in both images. The lesions are located within the corona radiata (cr) where multiple nerve fiber crossings occur; the fiber orientation map (Fig. 3L) reveals the intricate fiber organization in this region, with grayish-appearing color showing crossing fibers of multiple orientations. A closer look (Fig. 3M,N,P,Q) shows the multiple crossings of the fibers surrounding the lesion but more heterogeneously distributed fibers inside the lesion, reflecting the disorganization associated with local tissue destruction. This partial loss of axons was confirmed with LFB and neurofilament stains respectively on consecutive sections (Fig. 3O,R), showing patchy axon loss and spongiosis as well as axonal spheroids and dystrophic axons (fig. S3I,J).

Looking at more microscopic fiber changes in pathology, we compared the hippocampal pathways from a subject with epilepsy-associated hippocampal sclerosis to those from a healthy control, both for coronal sections at the level of the lateral geniculate nucleus, fig. S4. At a macroscopic level, the diminished volume of the sclerotic hippocampus, fig. S4A, was accompanied by a strong degeneration of pathways, with only a few crossings, over much of the hippocampus, fig. S4C. The perforant pathway (p), the principal source of entorhinal input to the hippocampus, overlying the medial subiculum, was almost completely degenerated, fig. S4D. On the other hand, the control hippocampus had multiple interwoven fibers across all subfields, fig. S4G, with an especially strong perforant pathway connecting the entorhinal cortex to the CA1, CA3, and dentate gyrus, fig. S4H.

Overall, ComSLI can reveal the organization of intact axonal fibers, despite demyelination, but does show loss of signal and loss of distributed axonal networks associated with neurodegeneration.

## Comparison to other fiber orientation techniques

We next sought to compare ComSLI to previously developed methods that yield fiber orientations using different mechanisms and physical phenomena.

**Fig. 4.**
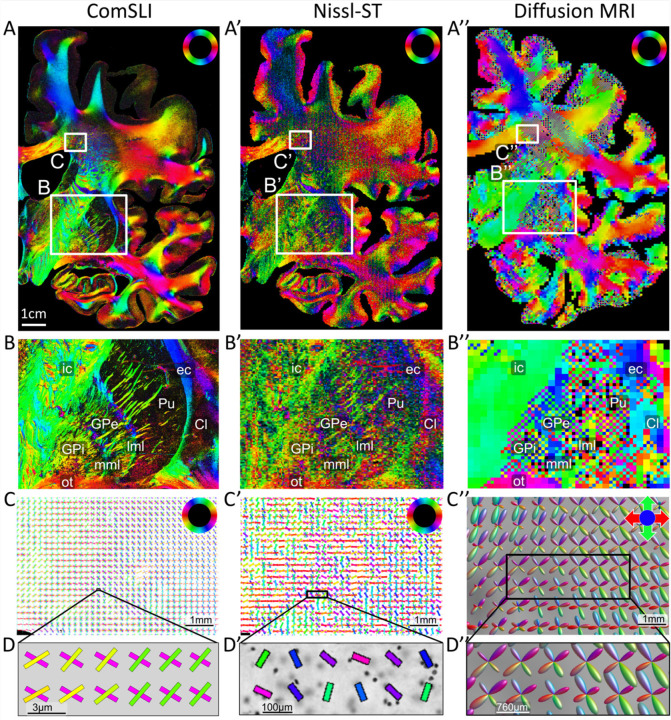
Comparison of ComSLI, Nissl-ST, and dMRI fiber orientations. ComSLI and Nissl-ST were performed on the same brain section (human coronal FFPE section, Nissl-stained with Cresyl violet, no. 3301) – the ComSLI measurement with 3μm pixel size, and Nissl-ST on the brightfield microscopy image with 1μm pixel size. Diffusion MRI was performed *in vivo* on a healthy volunteer with 0.76mm isotropic voxel size, and the orientation distribution functions were evaluated at a plane similar to the evaluated brain section (see Methods). (**A**) Color-coded fiber orientations depicted for each image pixel. The orientations were computed with *SLIX* (31) for ComSLI and dMRI, and with the provided code for Nissl-ST (26) using 15μm blur radius and a kernel size of 200×200μm. (**B**) Enlarged views of the fiber orientation maps for the bottom rectangular area marked in (A), featuring individual fiber bundles in the globus pallidus and putamen; annotated are internal capsule (ic), external capsule (ec), optic tract (ot), globus pallidus external segment (GPe), globus pallidus internal segment (GPi), putamen (Pu), medial and lateral medullary laminae (mml, lml), and claustrum (Cl). Nissl-ST orientations were computed with 15μm blur radius and a kernel size of 100×100μm. (**C**) Enlarged views of the fiber orientations for the top rectangular area in (A), featuring crossing fibers in the corona radiata. Nissl-ST orientations were computed with 15μm blur radius and a kernel size of 100×100μm. ComSLI fiber orientations were visualized as colored lines and overlaid on 66×66 pixels for better visualization and comparison; Nissl-ST orientations were overlaid on 2×2 pixels; dMRI orientation distribution functions were visualized with *mrtrix3*’s *mrview* (32). (**D**) Fiber orientations and distribution functions of 2×6 selected pixels, marked by the rectangular regions in (C), with orientation distributions displayed at the resolution of each method (3μm *vs*. 100μm *vs*. 760μm).

We first contrasted our results on whole human brain sections (Fig. 4, left) to those obtained using Nissl-based Structure Tensor (Nissl-ST) on the same section (Fig. 4, middle). Nissl-ST uses 2D structure tensor analysis to retrieve fiber orientation information in most white matter areas based on the anisotropic shape of Nissl-stained glial cells (26). We measured brain sections Nissl-stained with Cresyl violet (Fig. 4 and fig. S7), using the code and settings in (26) to derive in-plane fiber orientations. Figure S5 shows the resulting fiber orientations for different kernel sizes, from 200×200μm to 50×50μm, showing the 100μm kernel as the best balance between data quality and resolution. The fiber orientations are in overall agreement between Nissl-ST and ComSLI (Fig. 4A,A’), with ComSLI achieving better visualization of small features, such as fiber bundles in the putamen (Pu) and globus pallidus (GPe, GPi), the medial and lateral medullary laminae (mml, lml), and the external capsule (ec), Fig. 4B,B’. Moreover, ComSLI resolves multiple crossing fibers per 3μm pixel in a label-free manner, for example in the corona radiata region (Fig. 4C,D), with Nissl-ST providing single fiber orientation per 100μm pixel (Fig. 4D’).

We also compared our results to diffusion MRI-derived fiber orientations (Fig. 4, right) obtained from a high-resolution *in vivo* 18-hour diffusion MRI scan on the brain of a healthy volunteer (27). ComSLI fiber orientations were compared with those from the MR plane that best matched the measured brain sections (see Methods). The main nerve fiber orientation patterns of ComSLI and dMRI are mostly in agreement (Fig. 4A,A’’), with ComSLI unveiling distinct microscopic fiber orientation patterns, like the individual fiber bundles in the putamen and global pallidus or the medullary laminae (Fig. 4B), not resolved by dMRI *in vivo* (Fig. 4B’’) or *ex vivo* (28).

Comparing ComSLI, Nissl-ST and dMRI on other brain sections, such as the cell-body silver-stained *Jülich BigBrain* (24) section from Fig. 1C, yields similar conclusions (fig. S6).

Finally, 3D-Polarized Light Imaging (3D-PLI) provides micron-resolution fiber orientation maps of whole-brain sections (11–13). Our tests on the studied whole-brain FFPE sections showed significantly reduced sensitivity (fig. S7, right), compared to the high-sensitivity high-resolution maps it provides in 3D-PLI sections. This is possibly because an important part of myelin lipids resolves during alcohol dehydration (29), and water removal also distorts myelin periodicity (30), resulting in loss of its birefringence.

## Extension to tissues beyond brain

Finally, we aimed to retrieve fiber orientations from non-brain human tissues, given the multiple fiber types present, often in close proximity.

**Fig. 5.**
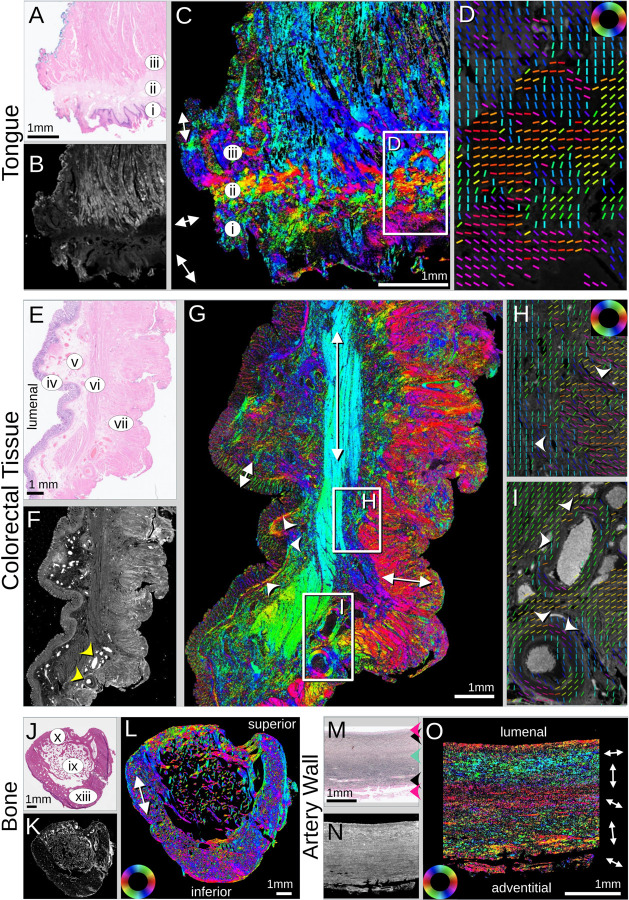
Non-brain samples measured with ComSLI. (A-D) Human tongue muscle. (**A**) Brightfield H&E image, 0.46μm/pixel; (i) epithelium, (ii) stroma, (iii) inner muscle layer. (**B**) Average scattering signal, 3μm/pixel. (**C**) ComSLI fiber orientation map, color-coded according to colorwheel in (D). (**D**) Zoomed-in area from (C) including different fiber layers; fiber orientations are displayed as colored lines for every 25^th^ pixel. (**E-I**) **Human colorectal tissue**. (**E**) Brightfield H&E image, 0.46μm/pixel; (iv) epithelium, (v) mucosa and submucosa, (vi) circular muscle, (vii) longitudinal muscle. (**F**) Average scattering signal, 3μm/pixel, with two blood vessels indicated by arrowheads. (**G**) ComSLI fiber orientation map, color-coded according to colorwheel in (H). Bidirectional arrows indicate main fiber orientation, arrowheads indicate the basal membrane. (**H**,**I**) Upper and lower zoomed-in areas from (G); fiber orientations are displayed as colored lines for every 40^th^ image pixel. Arrowheads indicate vertical and horizontal fiber orientations from circular and longitudinal muscle respectively (H), and blood vessels with fibers running circumferential (I). (**J**-**L**) **Human mandible**. (**J**) Brightfield H&E image, 0.46μm/pixel; (xiii) cortical bone on anterior side, (ix) trabecular (cancellous) bone, (x) cortical bone on tooth side. (**K**) Average scattering signal, 3μm/pixel. (**L**) ComSLI fiber orientation map, color-coded according to colorwheel. (**M**-**O**) **Elastic artery wall**. (**M**) Brightfield elastin-stained image, 0.46μm/pixel. (**N**) Average scattering signal, 3μm/pixel. (**O**) ComSLI fiber orientation map. Highlighted are fiber layers with different directions going from lumenal to adventitial surfaces. All samples are 4μm-thin FFPE sections and stained with either H&E (tongue, colorectal, mandible) or elastin staining (artery wall). Main fiber orientations in panels are indicated by bidirectional arrows.

We performed measurements on FFPE sections from different tissue types (Fig. 5): human tongue muscle, colorectal tissue, lower jaw bone (mandible), and arterial wall.

In the tongue, ComSLI reveals layers of differently oriented structures (Fig. 5A–D), like outer epithelium (i), underlying stroma (ii) with collagen fibers running parallel to the surface, and inner muscular layer (iii), which contains interwoven muscle fibers that enable tongue reshaping and multi-directional movement.

In colorectal tissue, Fig. 5E–I, orientations of multiple fiber types are retrieved: epithelial ciliae (iv) are typically oriented normal/radial to the surface, while the basal membrane microstructure is oriented parallel to the surface (arrowheads in Fig. 5G); the mucosal and submucosal fibers (v) have a complex architecture with various orientations; circular muscle fibers of the muscularis mucosa (vi) run from top to bottom (vertical bidirectional arrow in Fig. 5G and lower arrowhead in Fig. 5H); longitudinal fibers of the muscularis propria (vii) run perpendicular to the circular muscle fibers (horizontal bidirectional arrow in Fig. 5G and upper arrowhead in Fig. 5H). Blood vessels yield high scattering signal (arrowheads in Fig. 5F), with collagen fibers wrapping around vessel walls (arrowheads in Fig. 5I).

In mandible bone (Fig. 5J–L), the inner spongy bone (trabecular bone, ix) shows fibers mostly following trabecular microstructure (33). The U-shaped cortical bone (xiii) shows a dominant fiber orientation in the direction of the force applied during mastication (Fig. 5L, bidirectional arrow), while the superior region (x) exhibits more variable fiber orientations, distributing incoming loads to the rest of the jaw.

In artery wall, Fig. 5M–O, ComSLI reveals microscopic arrangements of fibers in different layers. While the brightfield image (Fig. 5M) indicates the fiber type (pinkish area dominated by collagen fibers, dark/black area by elastin fibers, grayish-greenish area by muscle fibers, indicated by arrowheads in corresponding color), the fiber orientation map (Fig. 5O) reveals distinct fiber directions in each layer, critical for artery mechanical properties (6): fibers run parallel to the surface in the inner, middle, and outer layers; in the layers in between, the fibers run perpendicular to the surface (bidirectional arrows).

ComSLI revealed directed structures of non-brain tissues both for stained and unstained sections (fig. S8), and yielded similar results across adjacent sections, for different section thicknesses (from 4μm to 15μm) and different steps of sample preparation, also for sections still in paraffin (fig. S8A,D,G).

## Conclusions

Most biological tissues are composed of various fibrous structures whose architecture determines tissue properties. Here, we show that microscopic fiber orientations can be retrieved using ComSLI from both new and archived paraffin-treated tissue sections prepared with different stains and protocols. The presented approach has the potential to make fiber orientation studies accessible to every laboratory: ComSLI is i) relatively low-cost, requiring only a lightsource (such as an LED light), a sample stage, and a micron-resolution camera, ii) fast, with a full measurement performed within seconds, iii) compatible with sections at virtually any stage of sample preparation. This enables microscopic exploration of fiber orientations in health and disease on sections readily available in thousands of laboratories and clinics worldwide.

Fiber orientation maps were retrieved from sections of various tissues including brain, tongue, colorectal, bone, and artery; the former was explored for human whole-brain, hippocampus, isocortex, pathology, and animal brain sections. ComSLI provided microscopic fiber maps, invisible with brightfield microscopy and inaccessible with other methods.

Compared to Nissl-ST, ComSLI provides higher resolution in a label-free manner, including multiple crossing fiber orientations per image pixel, and the possibility for out-of-plane orientation information (14–16). Diffusion MRI can image living human subjects tomographically but lacks cellular and microscopic details. Compared to diffusion MRI, ComSLI provides much higher resolution leading to visualization of small fiber tracts invisible to MRI. Also, ComSLI can be applied to any section, new or archived, at any sample preparation stage, even for sections still in paraffin. Given its relative insensitivity to demyelination, ComSLI can yield interesting pathologic insights when combined with myelin-sensitive microscopic methods such as 3D-PLI (11, 12), polarization-sensitive optical coherence tomography (34, 35), and X-ray scattering (20, 36, 37).

ComSLI enables retrospective studies that can shed light to changes in microscopic fiber orientations. In neuroscience, it can be applied to the multiple neurologic and psychiatric disorders with structural connectivity changes, such as Alzheimer’s disease (38), Parkinson’s disease (39), traumatic brain injury (40), stroke (41), multiple sclerosis (42), schizophrenia (43), epilepsy (44), or autism (45). It can be used across any brain circuitry and can validate *ex vivo* diffusion MRI orientations (21), especially in areas with small or highly interwoven fiber tracts such as the hippo-campus (13) or U-fibers (46), where sensitivity and high-resolution is key. In other organs, it can reveal the intricate organization of fibers that informs the structure and function of biological tissues such as collagen (47), actin/myosin (48), elastin (49), or the collagen fiber organization around tumors (50, 51) related to staging and malignancy, potentially allowing more accurate diagnosis and tailored treatment. It could also help create reference macroscopic maps of microscopic fiber orientation for entire organs, e.g., applied to and extending datasets such as the BigBrain datasets (24).

In this study, we only show in-plane fiber orientations, but the method also provides information of fiber inclination (14–16), and our future work will focus on quantifying out-of-plane angles. While measurements were performed in transmission mode through thin sections, we aim to extend measurements to back-scattering mode to analyze fiber orientations on surfaces without sectioning. Finally, ComSLI provides fiber orientations without distinguishing between nerve, muscle, collagen, or other fibers. Future studies will investigate the different scattering signatures from distinct fiber types to enable label-free, fiber-specific analyses.

In conclusion, ComSLI reveals the underlying microstructure of tissue sections including paraffin-embedded formalin-fixed (FFPE) sections, independent of staining and tissue preparation. This fast, simple to implement, and cost-effective technique can become a routine tool to provide micrometer fiber orientation mapping of histological tissue sections, opening new avenues in the investigation of the microarchitecture of any tissue, in health or disease.

## Materials and Methods

### Sample preparation

#### Whole human brain (BigBrain) sections

Two human brains, obtained within 24 hours after death and without neurological disorders, were fixed in 4% formaldehyde, dehydrated in increasing alcohol series (80%, 90%, 96%, 100% ethanol for at least one week each), embedded in a solvent (chloroform) for one week, and embedded in 57-60°C paraffin solution for two months. After hardening, the brains were coronally cut into 20 μm-thin sections from anterior to posterior with a large-scale microtome (Leica SM2500 Microtome) and mounted on a 37°C heat plate in gelatin solution. The sections were placed in a decreasing alcohol series to remove the paraffin and then placed in a staining solution to highlight neuronal cell bodies: the sections of the first brain (30 years old, male) were stained with silver following the protocol of Merker (52); the sections of the second brain (71 years old, male) were stained with Cresyl-violet. After staining, the sections were again dehydrated in increasing alcohol series, mounted/sealed on glass slides, and archived. The silver-stained sections were from the second BigBrain data set (53), 3D-reconstructed with the same spatial resolution of 20μm isotropic such as the original Jülich BigBrain (24), archived for 32 years, and section no. 3452 was selected for evaluation (Fig. 1 and fig. S6). The Cresyl-violet sections were archived for one and a half years, and sections no. 3301 (Fig. 4, left) and 2520 (fig. S7) were selected for evaluation. Body donors gave written informed consent for the general use of postmortem tissue used in this study for aims of research and education. The usage is covered by a vote of the ethics committee of the medical faculty of the Heinrich Heine University Düsseldorf, Germany (#4863).

#### 120-year-old myelin-stained brain section

The myelin-stained human brain section (Fig. 2B) comes from the brain collection of the Cécile and Oskar Vogt Institute for Brain Research, Heinrich Heine University Düsseldorf, Germany. The brain of a 25-year-old male was embedded in celloidin and stained according to Weigert’s iron hematoxylin myelin staining in 1904 (54).

#### Human hippocampus, cortex, and pathology brain sections

Four-millimeter thick specimens of formalin-fixed human brains were dehydrated in increasing ethanol steps (70% x2, 95% x2, 100% x3, 3.5hrs each step), cleared in xylene (3.5hrs x2), paraffin-embedded (3.5hrs x2), and sectioned into 5μm-thin sections. The sections were de-waxed and stained with different agents as indicated. The hippocampal sections in Fig. 2A and S1 were from a 89-year-old male with Alzheimer’s pathology, stained against microglia (CD163), Perl’s iron with Diaminobenzidine (DAB) enhancement, tau, and amyloid, with hematoxylin counterstain where indicated. Sections from brains with multiple sclerosis (80 years old, male, from temporal periventricular white matter and cortex) and leukoencephalopathy (43 years old, male, from periventricular white matter and cingulum) were stained with hematoxylin and eosin, luxol fast blue plus hematoxylin and eosin, and neurofilament (2F11) (Fig. 3 and S3). Hippocampal and visual cortex sections in fig. S2 were from a 60-year-old male stained with hematoxylin & eosin and a 67-year-old female stained with luxol fast blue respectively. The sclerotic hippocampal section in fig. S4 was from a 69-year-old female with epilepsy, the control was from a 66-year-old female with no neuropathologic abnormality. Specimens were acquired under Stanford ADRC IRB (Assurance nr. FWA00000935).

#### Mouse brain section

A female ~10-week-old C57BL/6 mouse (Jackson Laboratories) was housed in a temperature-controlled environment, with a 12-hour light/dark schedule and *ad libitum* food/water access. It was euthanized for the purposes of a different study (APLAC #32577) under anesthesia with 2–3% isoflurane followed by cardiac puncture and perfusion with 20mL phosphate buffered saline (PBS). The brain was harvested, kept in 4% paraformaldehyde (PFA) in PBS for 24 hours at 4°C, transferred to 10%, 20%, and 30% sucrose in PBS, embedded in Tissue-Tek O.C.T. in dry ice for 1 hour, and cut sagittally into 10μm sections using a cryotome (Leica CM1860). The sections were subsequently washed, mounted on a glass slide, intubated with Iba1 antibody (dilution 1:200), secondary antibody (goat anti-rabbit Cy3 1:200), and cover-slipped. A mid-sagittal section was selected for evaluation (Fig. 2F–I).

#### Pig brain section

A 4-week female Yorkshire pig (#2) was euthanized for a different study (Stanford APLAC protocol nr 33684), the brain was harvested, cut into 5-mm coronal slabs using a brain slicer, and a mid-frontal slab (#5) was paraffin-embedded, similar to the human pathologic specimen preparation above. The slab was cut in 10μm sections using a Leica HistoCore AUTOCUT microtome. After deparaffinization, a section (#127) was stained with hematoxylin and eosin and cover-slipped (Fig. 2J–M).

#### Human tongue, colorectal, bone, and artery wall sections

The non-brain tissue sections (Fig. 5 and S8) were obtained from a tissue archive at Erasmus Medical Center, Rotterdam, the Netherlands, approved by the Medisch Ethische Toetsing Commissie (METC) under number MEC-2023-0587. The tissue samples were obtained from patients during surgery. The bone sample was decalcified first using DecalMATE by Milestone Medical. Afterwards, all samples were fixed in 4% formaldehyde for 24 hours, dehydrated in increasing alcohol series (70%, 80%, 90%, 96%, 100% ethanol), treated with xylene, embedded in paraffin, and cut with a microtome (Leica RM2165) into 4μm-thin sections. The sections were placed in a decreasing alcohol series to remove the paraffin, mounted on glass slides, stained with hematoxylin and eosin (artery wall with Verhoeff-Van Gieson elastin staining), and then cover-slipped.

### Brightfield microscopy

The whole human brain sections were scanned with the TissueScope LE120 Slide Scanner by Huron Digital Pathology, Huron Technologies International Inc. The device measures in brightfield mode with 20X magnification and 0.74 NA, providing a pixel size of 0.4μm. The final images were stored with a pixel size of 1μm.

The hippocampus, cortex, pathology, and animal brain sections were scanned using an Aperio AT2 whole slide scanner with the ImageScope software and a 20X magnification, resulting in brightfield images with a pixel size of 0.5μm.

The stained non-brain microscopy slides were scanned using the Nanozoomer 2.0 HT digital slide scanner by Hamamatsu Photonics K.K., offering a 20X magnification and a pixel size of 0.46μm. The unstained non-brain microscopy slides were scanned using the Keyence VHX-6000 Digital Microscope (with VH-ZST objective, 20X), with a pixel size of 10μm.

### ComSLI

#### Whole human brain (silver-stained), hippocampus, cortex, pathology, and animal brain sections

Measurements were performed with a rotating lightsource and camera (cf. Fig. 1A), using a Flexacam C3 12 MP microscope camera (Leica) and a Navitar 12X Zoom Lens with a 0.67X Standard Adapter and a 0.5X Lens Attachment, with 4.25-9μm pixel size, as indicated in the figure captions. As lightsource, an ADJ Pinspot LED II was used, with 5.1cm diameter and 3.5° full-angle of divergence, oriented at ~45° with respect to the sample plane. A motorized specimen stage enabled the whole-human-brain section scanning in 8×5 tiles, all other brain sections were scanned at single tile. Images were acquired at 10° rotation steps (36 images/sample) with 125ms exposure time, except from the sections in paraffin that gave very strong scattering and were imaged with 7ms exposure time. Prior to the measurement, a 100mm diameter diffuser plate (Thorlabs) was measured for calibration (see below for calibration details).

#### Whole human brain (Cresyl-violet) and non-brain tissue sections

Measurements were similarly performed with a rotating lightsource and camera (cf. Fig. 1A), using a fiber-coupled LED lightsource consisting of an ultra-high power LED (UHP-FB-W50 by Prizmatix) with 400-750nm wavelength (peak at 443nm), 2-meter long step-index multimode silica (low OH) fiber patch cord (Thorlabs), a 25.4 mm diameter collimating optics (FCM1-0.5-CN by Prizmatix), and a 25.4 mm diameter engineered diffuser (beam shaper) for homogenizing the illumination (ED1-S20-MD by Thorlabs), yielding top-hat beam with 20° full-angle of divergence. The exposure time was adjusted manually per sample for maximizing the dynamic range of the captured signal while avoiding saturation (range: 50–100 ms). The lightsource was oriented at ~45° with respect to the sample and rotated with a motorized specimen stage (ZABER X-RSB060AD-E01-KX14A) in steps of 15° (24 images/sample). Images were taken with a 20 MP monochromatic CCD camera (BASLER acA5472-17um) and a Rodenstock Apo-Rodagon-D120 Lens, yielding a pixel size of 3μm (4μm optical resolution) and a field-of-view of 16×11mm^2^. A motorized specimen stage was used to perform whole-slide scanning. Prior to the measurement, a diffuser plate (DG100×100 N-BK7 ground glass diffuser, 1500 grit, Thorlabs) was measured for calibration.

#### 120-year-old myelin-stained brain section

The measurement was performed with a similar camera and lens as for the non-brain tissue sections (BASLER acA5472-17uc and Rodenstock Apo-Rodagon-D120), using an LED display instead of a focused lightsource (50×50cm^2^, 128×128 RGB-LEDs, Absen Polaris 3.9pro In/Outdoor LED Cabinet). The sample was illuminated by a green circle segment (9° azimuthal and polar widths, respectively) with an effective illumination angle of 47°, which was rotated in 15° steps. Images were taken with 10 seconds exposure time and a gain of 10, and 4 images were averaged per illumination angle to increase signal to noise.

#### Calibration

Prior to each measurement session, a diffuser plate was measured under similar conditions. The resulting images were blurred using a Gaussian blur with 100 pixels radius to homogenize diffuser defects. Subsequently, the blurred images of all angles were divided by the average of their maxima for normalization. These normalized diffuser images were used to calibrate the measured tissue images, aiming to account for the different light intensities across the field of view for each image: Each tissue image was divided by its corresponding normalized diffuser image of the same illumination angle.

#### Generation of fiber orientation and vector maps

Each calibrated image series from a ComSLI measurement was evaluated with the open-source software SLIX (31), which analyzes the position of scattering peaks to compute the fiber orientations and visualize them in in color-encoded maps, using multi-colored pixels and colored vector lines. Measurements with 15° azimuthal steps were processed without filtering. Measurements with 10° azimuthal steps were processed with Fourier low pass filter (40% cutoff frequency, 0.225 window width) before generating the parameter maps, as described in (15).

### Nissl-ST

The fiber orientation maps were computed in Matlab following the procedure described and code shared by Schurr & Mezer (26), using default settings and 100μm as kernel to compute the structure tensor (effective resolution).

### 3D-PLI

The 3D-PLI measurements were performed using the LMP3D microscope (Taorad GmbH, Germany), containing an evo4070MFLGEC (2048×2048) camera and a Nikon 4x (NA 0.2) lens, which achieves a pixel size of 1.85μm and an in-plane optical resolution of 2.2μm (determined by US-Airforce target). The sample was illuminated by linearly polarized light in 20° rotation angles and analyzed by a circular analyzer as described by Axer et al. (12). 3D-PLI FOM was computed on the supercomputer JURECA at Forschungszentrum Jülich (grant no. 28954).

### Diffusion MRI

The diffusion MRI dataset (27) is from a 30 year-old male who underwent 18 hours of diffusion MRI scanning in the MGH-USC 3T Connectom scanner using gSlider-SMS (55)(55). After manually identifying the MR plane that most closely matched the BigBrain histology sections, the entire dataset was rotated using FreeSurfer’s *freeview* and the b-vectors were rotated at the same angles (rotation angles for the Silver-stained section were −34° sagittal and 1.5° axial, for the Cresyl-violet −30.4° sagittal). Fiber responses and orientation distributions were computed using the *dwi2response* and *dwi2fod* functions in *mrtrix3* (32), using the multi-tissue, multi-shell algorithm (56), and visualized in *mrview*.(56) To generate whole-brain colormaps in Fig. 4 and S6, *mrtrix3*’s *sh2amp* function was used to probe fiber orientations at the coronal plane at 5° intervals, and colormaps were generated using SLIX (31).

## Supplementary Material

Supplement 1
